# Supplementary material for: Association between oral microbiome and five types of respiratory infections: a two-sample Mendelian randomization study in east Asian population
Source: Front Microbiol. 2024 Apr 10;15:1392473. doi: 10.3389/fmicb.2024.1392473 (PMC11039966; doi:10.3389/fmicb.2024.1392473)
Supplement: Supplementary file 1 [file Data_Sheet_1.zip › Supplementary Materials/MR plots of saliva/Chronic sinusitis/s__Streptococcus_mitis_AT_mgs_1319/forest.pdf]

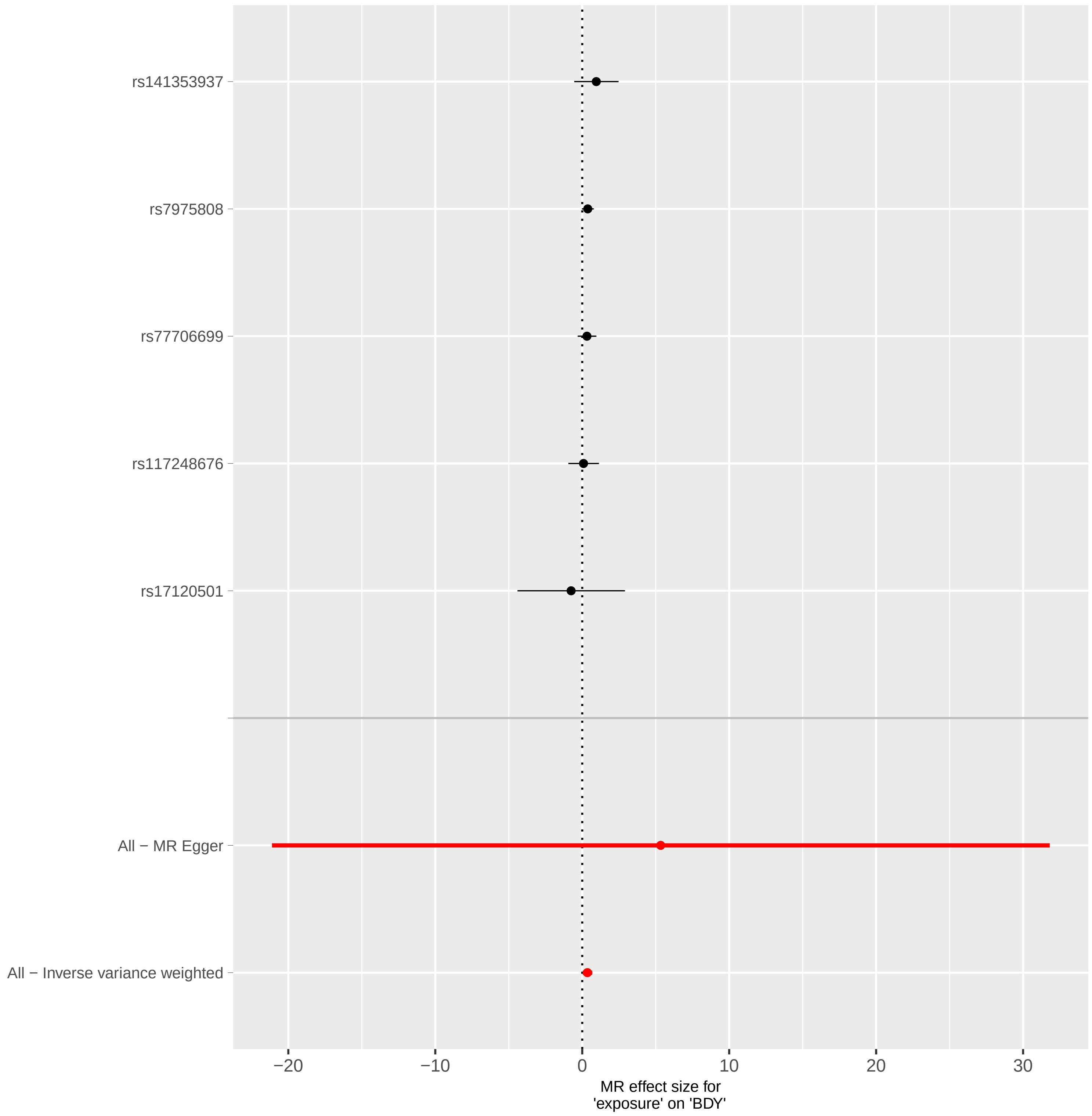
rs141353937
rs7975808
rs77706699
rs117248676
rs17120501
All − MR Egger
All − Inverse variance weighted
−20
−10
0
10
20
30
MR effect size for
'exposure' on 'BDY'
